# The role of non‐genomic actions of progesterone and its membrane receptor agonist in ovarian cancer cell death

**DOI:** 10.1002/cnr2.1934

**Published:** 2023-11-27

**Authors:** Takahiro Koyanagi, Yasushi Saga, Yoshifumi Takahashi, Kohei Tamura, Takahiro Yoshiba, Suzuyo Takahashi, Akiyo Taneichi, Yuji Takei, Hiroaki Mizukami, Hiroyuki Fujiwara

**Affiliations:** ^1^ Department of Obstetrics and Gynecology School of Medicine, Jichi Medical University Tochigi Japan; ^2^ Division of Genetic Therapeutics Center for Molecular Medicine, Jichi Medical University Tochigi Japan

**Keywords:** cell death, genomics, medroxyprogesterone acetate, ovarian cancer, progesterone, progesterone receptor

## Abstract

**Background:**

Progesterone therapy is a relatively inexpensive treatment option for endometrial and breast cancers, with few side effects. Two signaling pathways usually mediate the physiological effects of progesterone, namely genomic and non‐genomic actions. Genomic action occurs slowly via the nuclear progesterone receptor (PR), whereas the membrane progesterone receptor (mPR) induces rapid non‐genomic action.

**Aims:**

We investigated the effects of progesterone and various PR agonists on ovarian cancer cells.

**Methods and Results:**

PR expression of six serous ovarian cancer cell lines was examined by western blotting, and mPR expression was examined by reverse transcription‐quantitative polymerase chain reaction (RT‐qPCR). PR‐negative and mPR‐positive ovarian cancer cells were exposed to progesterone and seven types of PR agonists (medroxyprogesterone acetate [MPA], dehydroepiandrosterone, dienogest, levonorgestrel, drospirenone, pregnenolone, and allopregnanolone) at 10–400 μM, and viable cell counts after exposure for 30 min were measured using the water‐soluble tetrazolium (WST‐1) assay. Ovarian cancer cell lines were exposed to 100 μM progesterone, and the expression of BAX, a pro‐apoptotic protein, after 1–5 min was examined by western blotting.

Western blotting detected no PR expression in the six serous ovarian cancer cell lines. In contrast, RT‐qPCR detected mPR expression in all six serous ovarian cancer cell lines. Progesterone and MPA‐induced cell death in all tested ovarian cancer cell lines in a concentration‐dependent manner, whereas no effect was observed for other PR agonists. Western blotting revealed that pro‐apoptotic protein BAX expression occurred 1 min after exposure to progesterone, suggesting that the cytocidal effects are mediated by rapid non‐genomic action.

**Conclusion:**

Progesterone and MPA exhibited a rapid cytocidal effect on PR‐negative ovarian cancer cells through non‐genomic action. Progesterone and MPA could be novel adjuvant therapies for ovarian cancer.

## 1 INTRODUCTION

In the United States, ovarian cancer is the fifth leading cause of cancer‐related deaths in women. It is estimated that approximately 20 000 women will develop ovarian cancer and approximately 13 000 women will die of this disease every year.^1^ More than half of the patients are diagnosed with progressive disease with peritoneal dissemination and massive ascites because the early stage of ovarian cancer is asymptomatic. The standard treatments for advanced ovarian cancer are debulking surgery and adjuvant chemotherapy with platinum and taxane, which initially achieve a complete response in approximately 80% of patients. However, the antitumor effects are usually transient, and many patients experience abdominal recurrence with reduced chemosensitivity, ultimately leading to death due to disease progression.^2^, ^3^ Recently, several novel molecular targeted agents, including bevacizumab and poly adenosine diphosphate‐ribose polymerase (PARP) inhibitors, have been applied clinically.^4^ Although these drugs exert some antitumor effects, adverse side effects and economic burdens are challenges that need to be addressed. Therefore, more cost‐effective novel therapeutic strategies are required.

Progesterone is a steroid hormone that is primarily synthesized in the corpus luteum of the ovary. It forms a complex with the intracellular progesterone receptor (PR) proteins. This complex binds to the DNA promotor sites in the nucleus, thereby regulating the expression of various genes.^5^ For cancer therapy, progesterone is applied to endometrial and breast cancers as an inexpensive treatment option with few side effects.^6^, ^7^ In 2003, the membrane progesterone receptor (mPR) family was identified by Zhu et al.^8^ These mPRs exert rapid non‐genomic action, whereas the commonly known genomic action occurs more slowly through the nuclear PR.^9^

Our group previously reported that the incidence of the p53 signature in fallopian tubes was significantly lower in pregnant and parous women.^10^ Another report suggested that progesterone from follicular fluid during ovulation induces necroptosis in p53 mutated tubal epithelial cells and prevents carcinogenesis.^11^ Progesterone may prevent early‐phase serous carcinogenesis and suppress ovarian cancer cell growth.

In the present study, we evaluated the antitumor effects of progesterone and its derivatives (PR agonists) in ovarian cancer cell lines to obtain some clues for developing a novel therapeutic strategy.

## 2 MATERIALS AND METHODS

### 2.1 Cell lines and culture

The human ovarian serous adenocarcinoma cell lines SHIN‐3,^12^ KOC‐2S,^13^ and TU‐OS‐4^14^ were provided by the respective establishers. The human ovarian serous adenocarcinoma cell lines OVSAHO and OVKATE were purchased from the Japanese Collection of Research Bioresources (JCRB; Osaka, Japan). The human ovarian cancer cell line SKOV‐3 was purchased from the American Type Culture Collection (ATCC, Manassas, VA, USA). The human breast adenocarcinoma cell line MCF‐7 was purchased from RIKEN BioResource Research Center (Tsukuba, Japan). The cells were cultured in Dulbecco's Modified Eagle Medium/F12 (DMEM/F12; Thermo Fisher Scientific, Inc., Waltham, MA, USA) supplemented with 10% heat‐inactivated fetal bovine serum (FBS; Sigma‐Aldrich; Merck KGaA, Darmstadt, Germany) and 1% penicillin/streptomycin (Thermo Fisher Scientific, Inc.) at 37°C in a humidified atmosphere under 5% CO_2_.

### 2.2 Western blot analysis

To detect PR‐α and PR‐β, each tumor cell line (SHIN‐3, KOC‐2S, OVKATE, OVSAHO, TU‐OS‐4, and SKOV‐3) was seeded onto 6‐well plates at 2 × 10^5^ cells/well and incubated for 24 h. Also, to examine the effect of progesterone on BAX expression, SHIN‐3, KOC‐2S, and OVSAHO cells (2 × 10^5^/well) were seeded onto 6‐well plates, incubated for 24 h, then cultured in a medium containing 10% fetal calf serum with 100 μM progesterone (FUJIFILM Wako Pure Chemical Co., Osaka, Japan) for 1–5 min. Cells were lysed using lysis buffer (1% NP‐40, 150 mM NaCl, 50 mM Tris–HCl; pH 8.0), and extracted proteins were mixed with 1% sodium dodecyl sulfate (SDS) sample buffer (10 mM Tris–HCl [pH 7.5], 150 mM NaCl, 1% SDS, EDTA‐free protease inhibitor cocktail; Roche, Basel, Switzerland), separated by electrophoresis using 5%–10% polyacrylamide gels, and transferred onto polyvinylidene fluoride (PVDF) membranes (Merck KGaA, Darmstadt, Germany). Membranes were left at room temperature for 1 h in PVDF Blocking Reagent for Can Get Signal® (Toyobo Life Science, Osaka, Japan), washed three times using Tris‐buffered saline‐Tween‐20 (TBS‐T), and incubated overnight with the following antibodies at room temperature in Can Get Signal® Immunoreaction Enhance Solution 1 (Toyobo Life Science): Anti‐PR mouse monoclonal antibody (cat. no. sc‐810; 1:100; Santa Cruz Biotechnology, Inc., Dallas, TX, USA) and anti‐BAX (D2E11) rabbit monoclonal antibody (cat. no. 5023; 1:1000; Cell Signaling Technology, Danvers, MA, USA) and anti‐actin rabbit polyclonal antibodies (cat. no. A2066; 1:1000; Sigma‐Aldrich; Merck KGaA). After incubation, the membranes were washed three times with TBS‐T and incubated with peroxidase‐labeled anti‐mouse or anti‐rabbit antibodies (1:1000; GE Healthcare Japan, Tokyo, Japan) in Can Get Signal® Immunoreaction Enhance Solution 2 (Toyobo Life Science) at room temperature for 1 h. The membranes were then washed three times with TBS‐T, incubated with ECL prime western blotting detection reagent (GE Healthcare Japan), and imaged using a cooled charge‐coupled device system (LAS‐4000 mini; GE Healthcare Japan). The relative protein expression level was compared with that of actin as 1.0 using ImageJ software (NIH).

### 2.3 Quantitative reverse transcription PCR (RT‐qPCR)

Cellular mRNA was extracted using the RNeasy Mini Kit (Qiagen, Valencia, CA, USA) according to the manufacturer's instructions. RT‐qPCR was performed using the Thermal Cycler Dice Real‐Time System II (TAKARA BIO) following the manufacturer's instructions. PCR was conducted using 40 cycles of heating at 95°C for 15 s, 58°C for 15 s, and 72°C for 20 s. The mRNA levels of target genes were determined relative to the fluorescence signal of glyceraldehyde‐3‐phosphate dehydrogenase (GAPDH). The primer sequences were as follows: GAPDH: forward: 5′‐ACCACAGTCCATGCCATCAC‐3′, reverse: 5′‐CATCACGCCACAGTTTCCCG‐3′; mPRα: forward: 5′‐CGCTCTTCTGGAAGCCGTACATCTATG‐3′, reverse: 5′‐CAGCAGGTGGGTCCAGACATTCAC‐3′; mPRβ: forward: 5′‐ CTTCCGGGAGCCTTACATCC‐3′, reverse: 5′‐AAAGGCCCAGAATCGCAAGA‐3′; mPRγ: forward: 5′‐CAGCTGTTTCACGTGTGTGTGATCCTG‐3′, reverse: 5′‐ GCACAGAAGTATGGCTCCAGCTATCTGAG‐3′; mPRδ: forward: 5′‐ GGTGTTCTGGGAAGATGGCA‐3′, reverse: 5′‐TCTGGAAGGAGCTGAGGACA‐3′; and mPRε: forward: 5′‐CGTGGAGTGCTTCATCCTGT‐3′, reverse: 5′‐ AAGTTGAGCGTCTCGTTGGT‐3′.

### 2.4 Colorimetric assay

The sensitivity of tumor cells to progesterone (P4, FUJIFILM Wako Pure Chemical Co., Osaka, Japan), medroxyprogesterone acetate (MPA, Selleck Biotech, Tokyo, Japan), dehydroepiandrosterone sulfate (DHEA, MedChemExpress, Monmouth Junction, NJ, USA), dienogest (DNG, Cayman Chemical, Ann Arbor, MI, USA), levonorgestrel (LNG, Sigma‐Aldrich; Merck KGaA), drospirenone (DRSP, Cayman Chemical), pregnenolone (PREGN, MedChemExpress), and allopregnanolone (Allo, (FUJIFILM Wako Pure Chemical Co.) was measured using a colorimetric assay (Premix WST‐1 Cell Proliferation Assay System; Takara Bio Inc., Tokyo, Japan). Tumor cells were exposed to each drug at concentrations of 10–400 μM for 30 min. The viable cell count measured using the colorimetric assay is presented as a percentage ratio to the count of the control untreated with drugs.

## 3 RESULTS

### 3.1 PR expression

**FIGURE 1:**
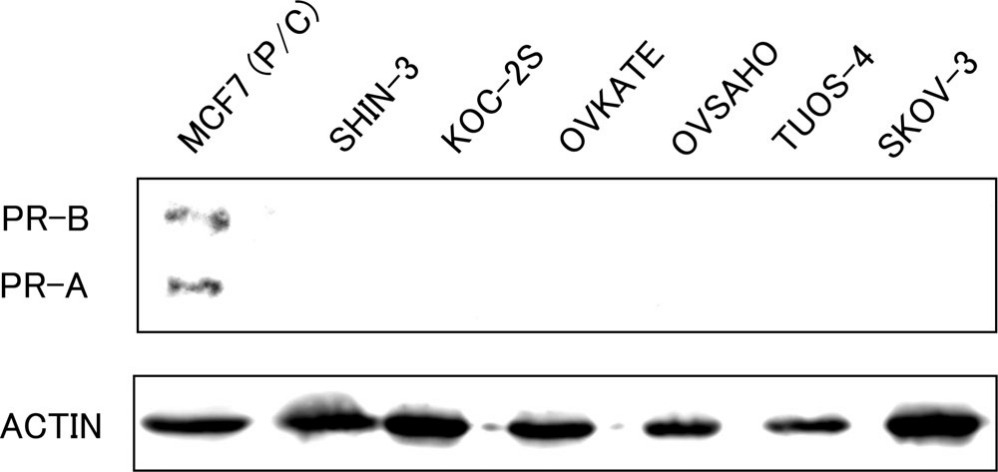
Western blotting of progesterone receptor (PR)‐α and PR‐β in ovarian serous carcinoma cell lines, SHIN‐3, KOC‐2S, OVKATE, OVSAHO, TU‐OS‐4, and SKOV‐3. Control cell line MCF‐7 clearly expressed PR‐α and PR‐β. No expression of PR was identified in the six serous ovarian cancer cell lines. PR, progesterone receptor; P/C, positive control.

Firstly, PR expression was evaluated using western blot analysis. As shown in Figure 1, PR expression was not detected in any of the tested ovarian cancer cell lines, suggesting that the genomic action does not occur in these cells. The human breast adenocarcinoma cell line MCF‐7 was used as the positive control.

### 3.2 mPR expression

**FIGURE 2:**
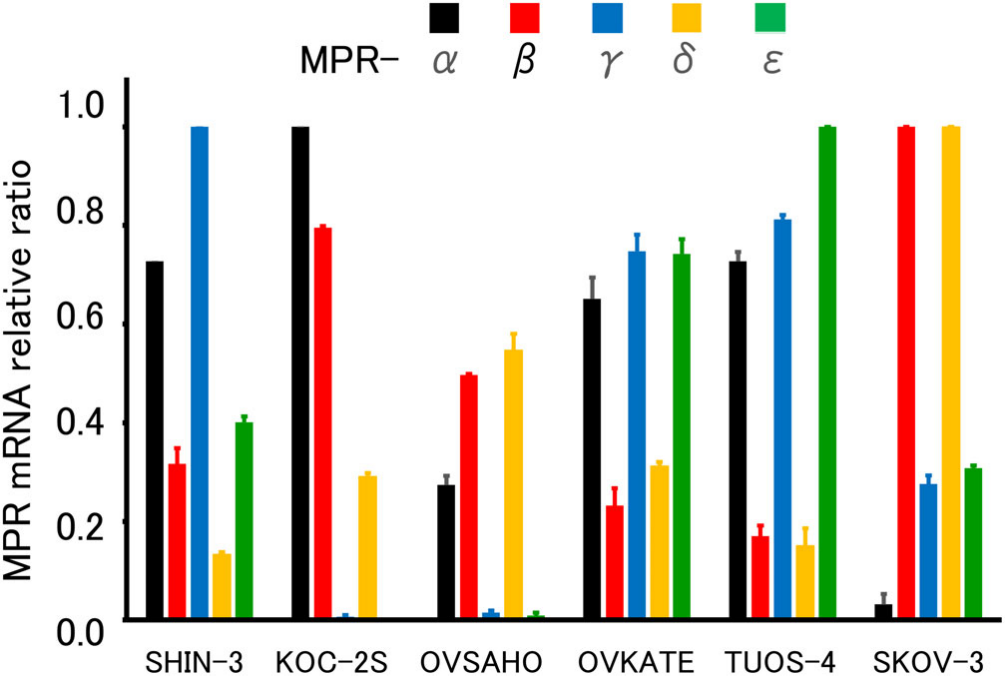
Confirmation of the expression of membrane progesterone receptor (mPR). mPR expression was identified in all six ovarian cancer cell lines by RT‐qPCR. mPR, membrane progesterone receptor.

Next, the expression of five types of mPRs (α, β, γ, δ, and ε) was evaluated using RT‐qPCR. All tested ovarian cancer cell lines expressed various mRNAs with various values (Figure 2). These results suggest that progesterone could show non‐genomic actions through mPRs in these ovarian cancer cells.

### 3.3 Effects of progesterone and PR agonists

**FIGURE 3:**
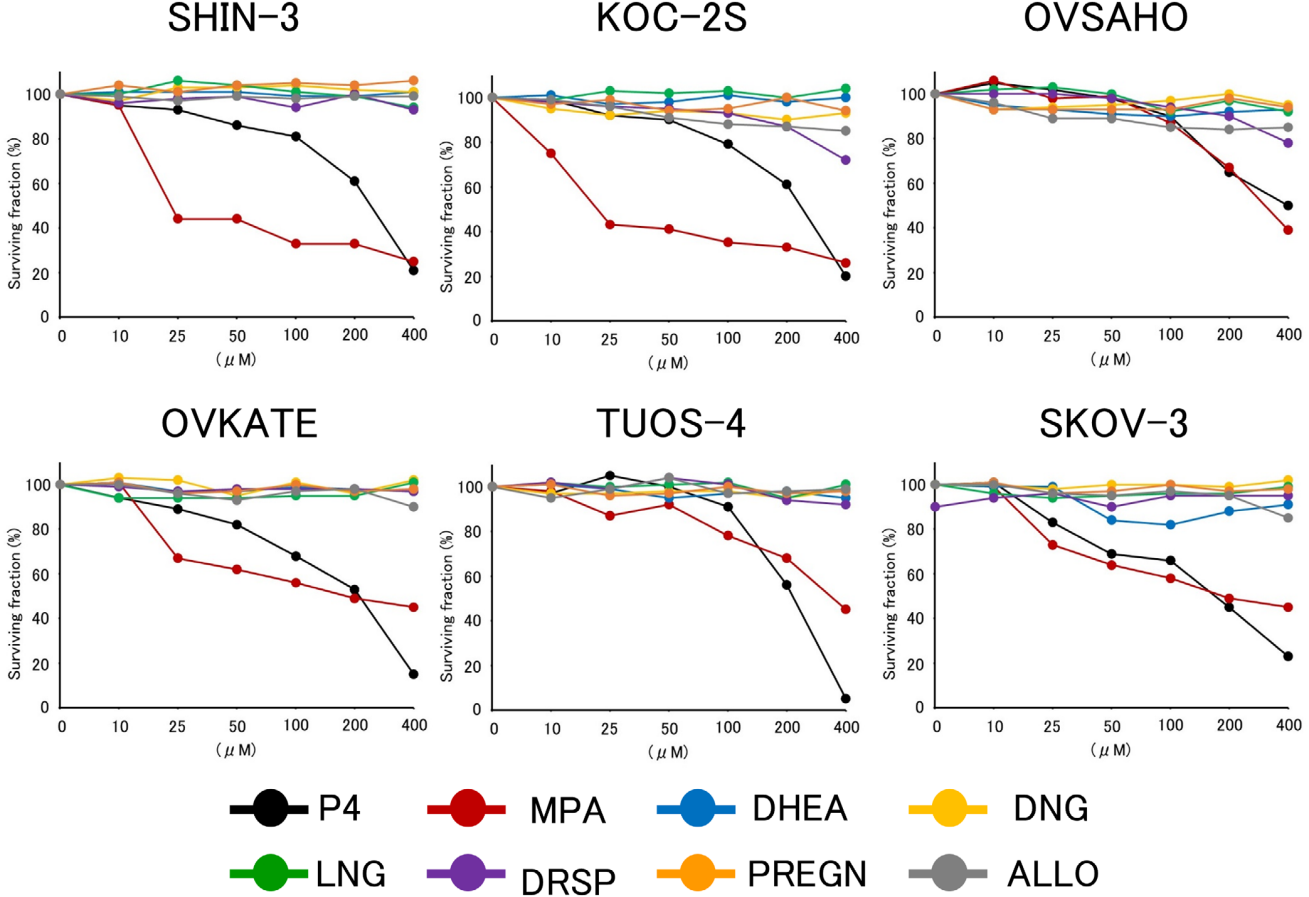
Cytocidal effects of progesterone and PR agonists on ovarian cancer cell lines. The viable cell count was measured by the WST‐1 assay after 30 minutes' exposure to each drug at the concentrations of 10–400 μM. The data were presented as a percentage ratio to the count of the control untreated with drugs. P4, progesterone; MPA, medroxyprogesterone acetate; DHEA, dehydroepiandrosterone sulfate; DNG, dienogest; LNG, levonorgestrel; DRSP, drospirenone; PREGN, pregnenolone; ALLO, allopregnanolone.

The cytocidal effect of progesterone and PR agonists was examined in each ovarian cancer cell line. As shown in Figure 3, progesterone and one of its derivatives (MPA) induced distinct cell death in all tested ovarian cancer cell lines in a concentration‐dependent manner. However, PR agonists other than MPA did not exhibit cytocidal effects on any ovarian cancer cell lines.

### 3.4 BAX expression

**FIGURE 4:**
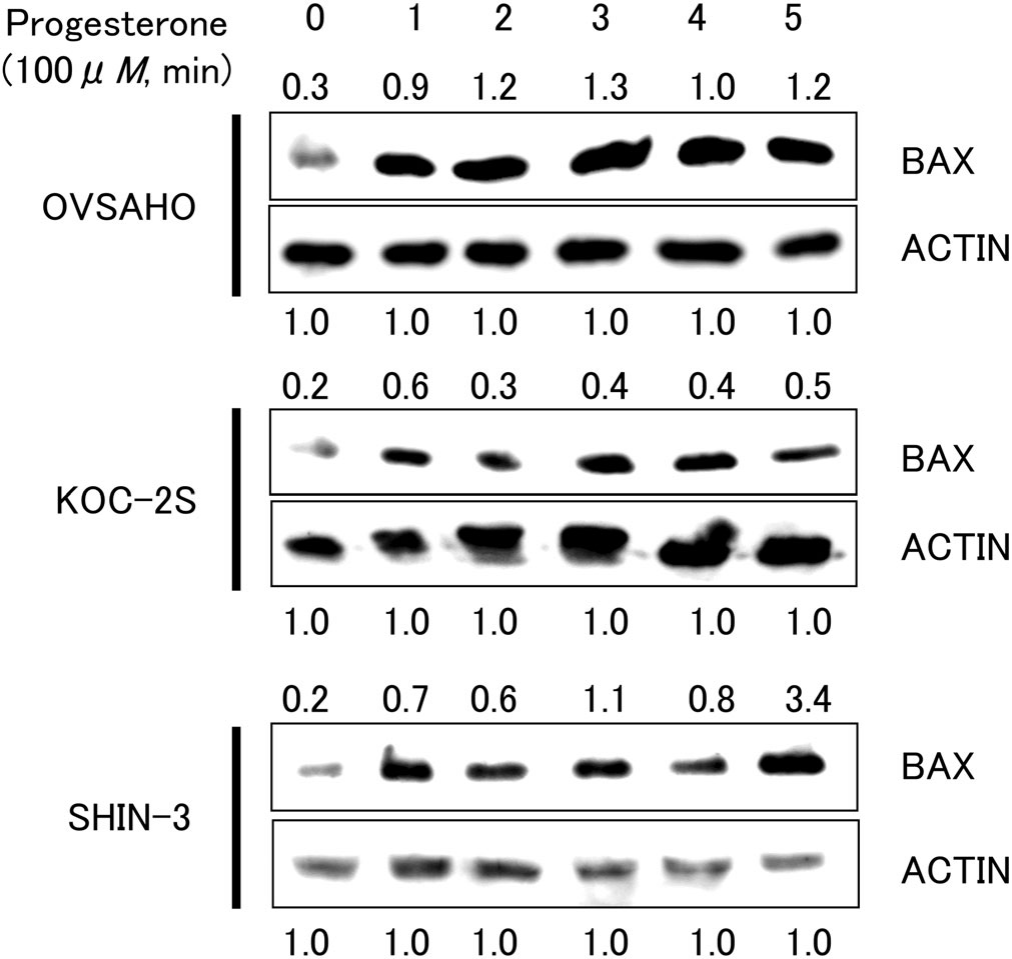
Western blotting of one of the pro‐apoptotic proteins, BAX after exposure to progesterone. Each ovarian cancer cell line was exposed to 100 μM of progesterone for 1–5 min. Upregulation of BAX protein was noted 1 min after exposure to progesterone. Each number indicates the relative protein expression level compared with Actin as 1.0.

The expression of one of the pro‐apoptotic proteins, BAX, was evaluated by western blotting. BAX protein expression was detected at a position corresponding to a molecular weight of 20 kDa in all tested cell lines exposed to progesterone for 1–5 min (Figure 4). Upregulation of BAX protein was noted 1 min after exposure to progesterone, suggesting that this prompt response may result from the non‐genomic action of progesterone via the mPR.

## 4 DISCUSSION

In the present study, nuclear PR was not expressed in the six ovarian serous carcinoma cell lines. In contrast, all six ovarian cancer cell lines expressed various mPRs. Progesterone and one of its derivatives, MPA, exerted concentration‐dependent cytocidal effects. No cytotoxic effects were observed for the other PR agonists. In addition, the expression of the pro‐apoptotic protein BAX increased 1 min after exposure to progesterone.

PR is physiologically expressed in organs, such as the mammary glands, endometrium, and ovaries. Regarding malignancies, PR is expressed in breast and endometrial cancers, whereas most ovarian cancers lack its expression.^15^ In this study, no PR expression was identified in all the examined ovarian cancer cell lines.

mPRs are membrane‐bound receptors with high affinity and specificity for progesterone and adiponectin. mPRs induce non‐genomic actions by activating non‐canonical intracellular signaling pathways. Genomic action via transcriptional regulation by activated nuclear PR occurs slowly, whereas non‐genomic action via mPR is rapidly induced. There are five types of mPRs, namely mPR‐α, β, γ, δ, and ε.^9^ mPR expression in ovarian cancer was previously reported.^16^ mPR expression was identified in all six cell lines examined in this study.

Progesterone and MPA displayed concentration‐dependent cytocidal effects. However, no cytocidal effects were observed for the other PR agonists. This cytocidal effect appears to be mediated by non‐genomic actions via mPRs because it was observed in PR‐negative and mPR‐positive ovarian cancer cells within a short time (30 min). Pang et al.^17^ demonstrated that PR agonists (MPA not included) possess a significantly lower binding affinity for mPR than progesterone. Most PR agonists may not affect ovarian cancer cell survival because of their low binding affinity for mPR.

BAX is a member of the Bcl‐2 family that accelerates apoptosis. Recently, BAX has been reported to be associated with other programmed cell death pathways, including pyroptosis and ferroptosis.^18^, ^19^ Charles et al.^16^ demonstrated that 24 h progesterone stimulation induced BAX overexpression in PR‐negative and mPR‐positive ovarian cancer cells using comprehensive genetic analysis with a qPCR super array. In the present study, BAX expression was rapidly induced 1 min after progesterone exposure. This prompt response may result from the non‐genomic action of progesterone via the mPR.

Recently, novel molecular targeted agents, including bevacizumab and PARP inhibitors, have been applied in clinical practice, especially in maintenance therapy for ovarian cancer.^20^ Although these drugs exhibit antitumor effects to some extent, side effects such as hypertension, proteinuria, and gastrointestinal perforation are associated with bevacizumab treatment, while severe anemia, thrombocytopenia, and renal dysfunction are associated with PARP inhibitors. Another challenge is the economic burden owing to the high cost of these novel drugs. Progesterone and MPA are clinically used for threatened premature birth, and their cost is relatively low.^21^ Therefore, progesterone and its derivatives are safer and less expensive than chemotherapeutic agents and molecular targeted drugs.

In this research, progesterone and MPA exerted cytocidal effects against ovarian cancer cells at concentrations of 10–400 μM. This concentration range is much higher than those used in pregnant women or patients with cancer taking MPA, and approximates the concentration in follicular fluid under physiological conditions.^22^ Progesterone is rapidly metabolized in vivo by various hydroxylases synthesized in the liver. The serum concentration of progesterone increases in patients with congenital adrenal hyperplasia who lack these hydroxylases.^23^ When applying progesterone and its derivatives in ovarian cancer treatment, a combination of some hydroxylase inhibitors might be useful. Further investigations including in vivo experiments are required to establish a novel therapeutic strategy targeting mPRs.

In conclusion, MPA and progesterone exhibited a rapid cytocidal effect on PR‐negative ovarian cancer cells through non‐genomic action. Progesterone and its derivative MPA could be new treatment modalities for ovarian cancer.

## AUTHOR CONTRIBUTIONS

**Takahiro Koyanagi:** Funding acquisition (lead); investigation (equal); writing – original draft (equal); writing – review and editing (equal). **Yasushi Saga:** Investigation (equal); methodology (lead); writing – original draft (equal); writing – review and editing (equal). **Yoshifumi Takahashi:** Investigation (supporting). **Kohei Tamura:** Investigation (supporting). **Takahiro Yoshiba:** Investigation (supporting). **Suzuyo Takahashi:** Investigation (supporting). **Akiyo Taneichi:** Investigation (supporting). **Yuji Takei:** Investigation (supporting). **Hiroaki Mizukami:** Writing – review and editing (supporting). **Hiroyuki Fujiwara:** Writing – review and editing (supporting).

## CONFLICT OF INTEREST STATEMENT

The authors declare no conflicts of interests.

## Supporting information


**Data S1:** Raw data images of western blotting with a marker ladder (Figures 1 and 4) are provided in the supporting information.

## Data Availability

The data that support the findings of this research are available from the corresponding author upon reasonable request.
