# Supplementary material for: The role of non‐genomic actions of progesterone and its membrane receptor agonist in ovarian cancer cell death
Source: Cancer Rep (Hoboken). 2023 Nov 27;7(1):e1934. doi: 10.1002/cnr2.1934 (PMC10809274; doi:10.1002/cnr2.1934)
Supplement: Supplementary file 1 — Data S1: Raw data images of western blotting with a marker ladder (Figures 1 and 4) are provided in the supporting information. [file CNR2-7-e1934-s001.ppt]

## Slide 1
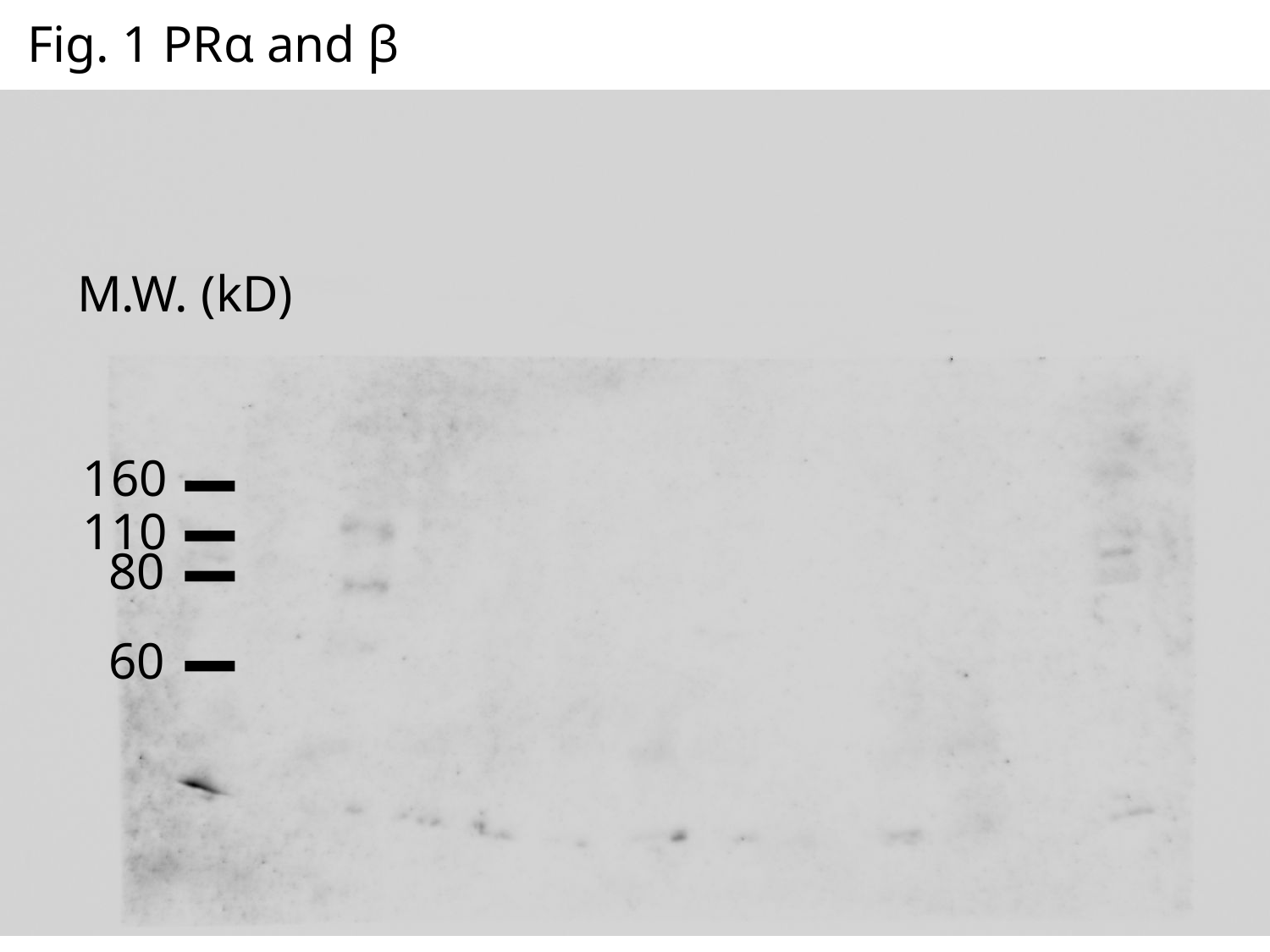

Fig. 1 PRα and β
M.W. (kD)
160
110
80
60

## Slide 2
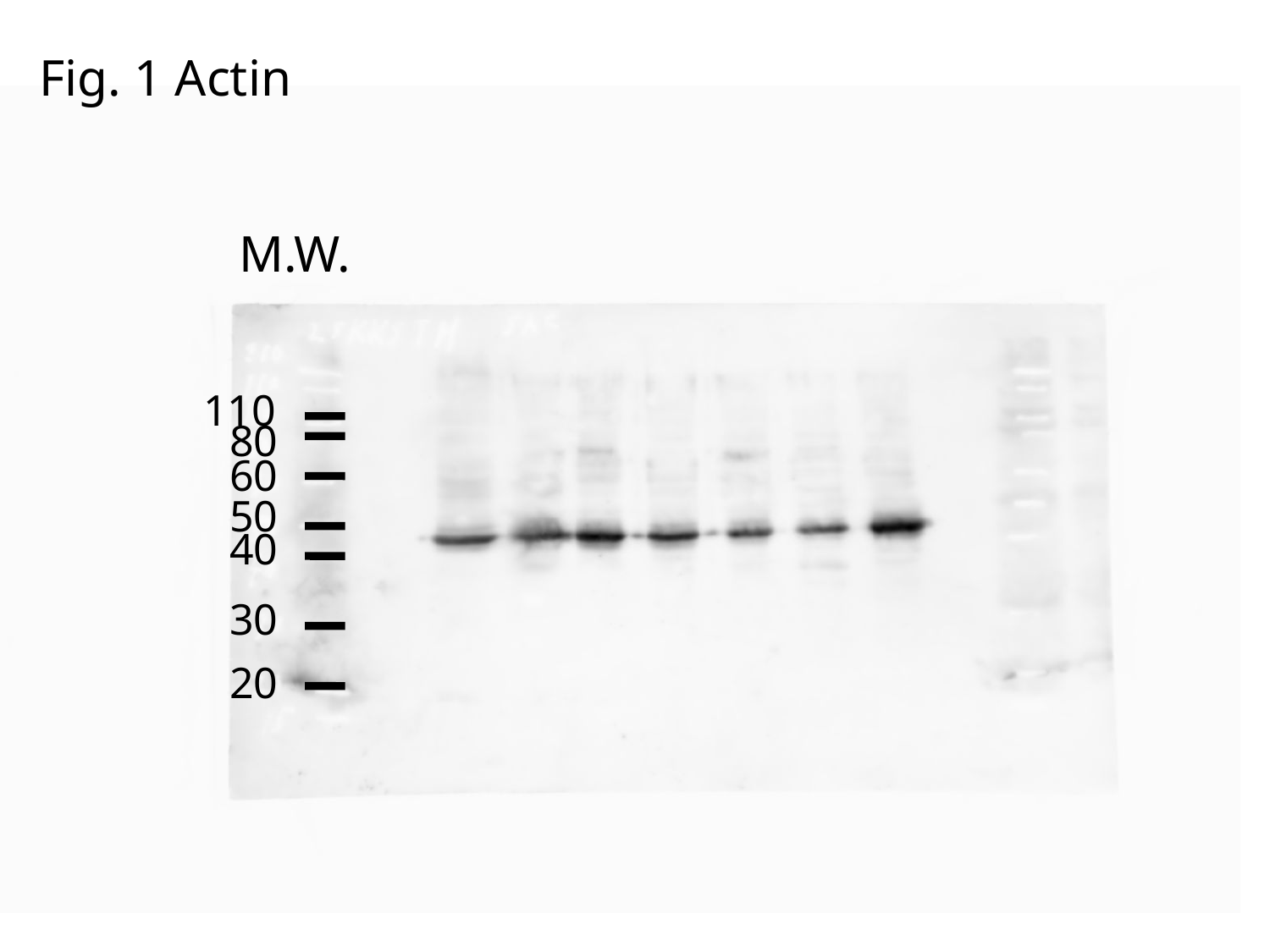

Fig. 1 Actin
M.W.
110
80
60
50
40
30
20

## Slide 3
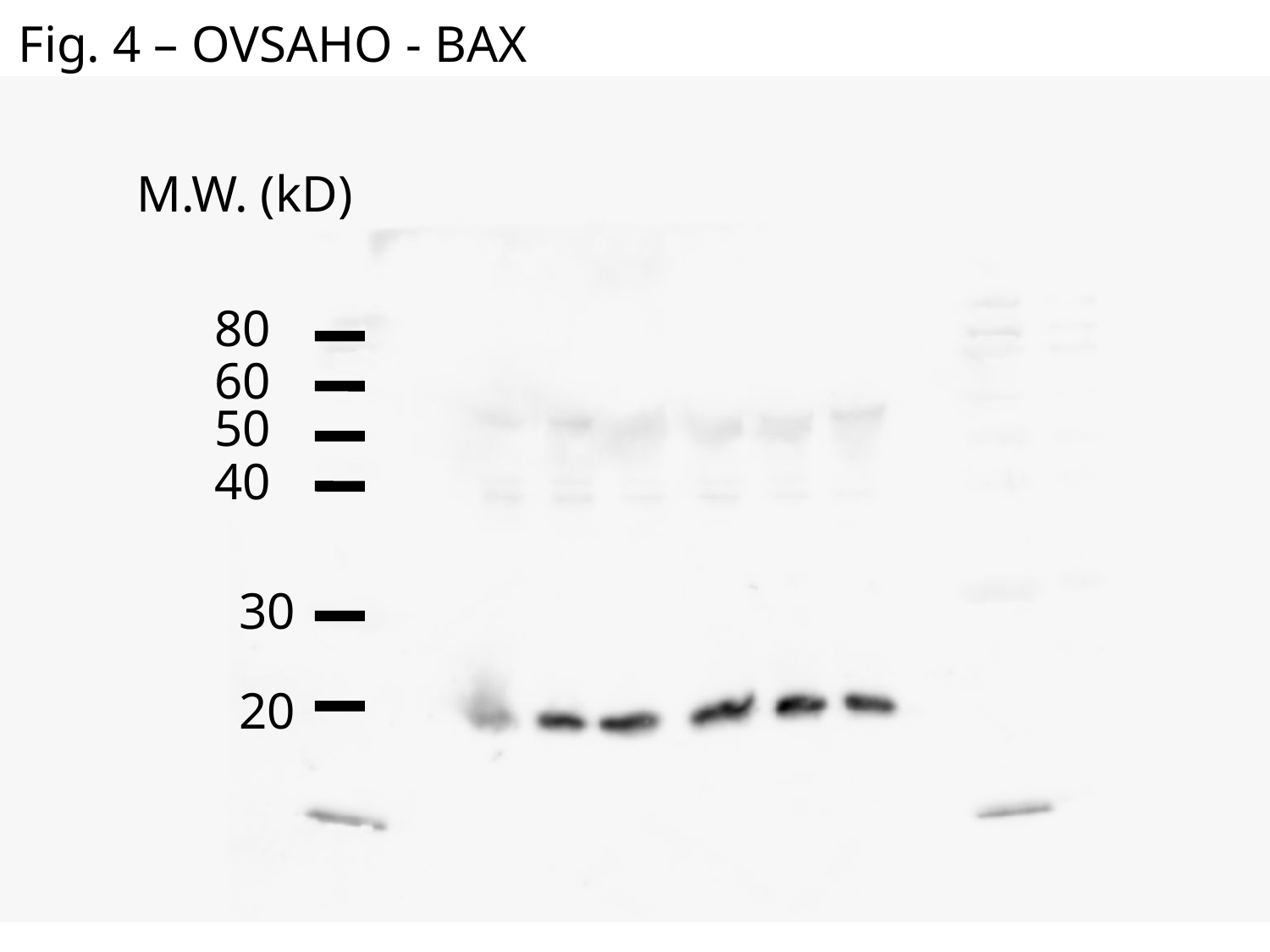

Fig. 4 – OVSAHO - BAX
M.W. (kD)
80
60
50
40
30
20

## Slide 4
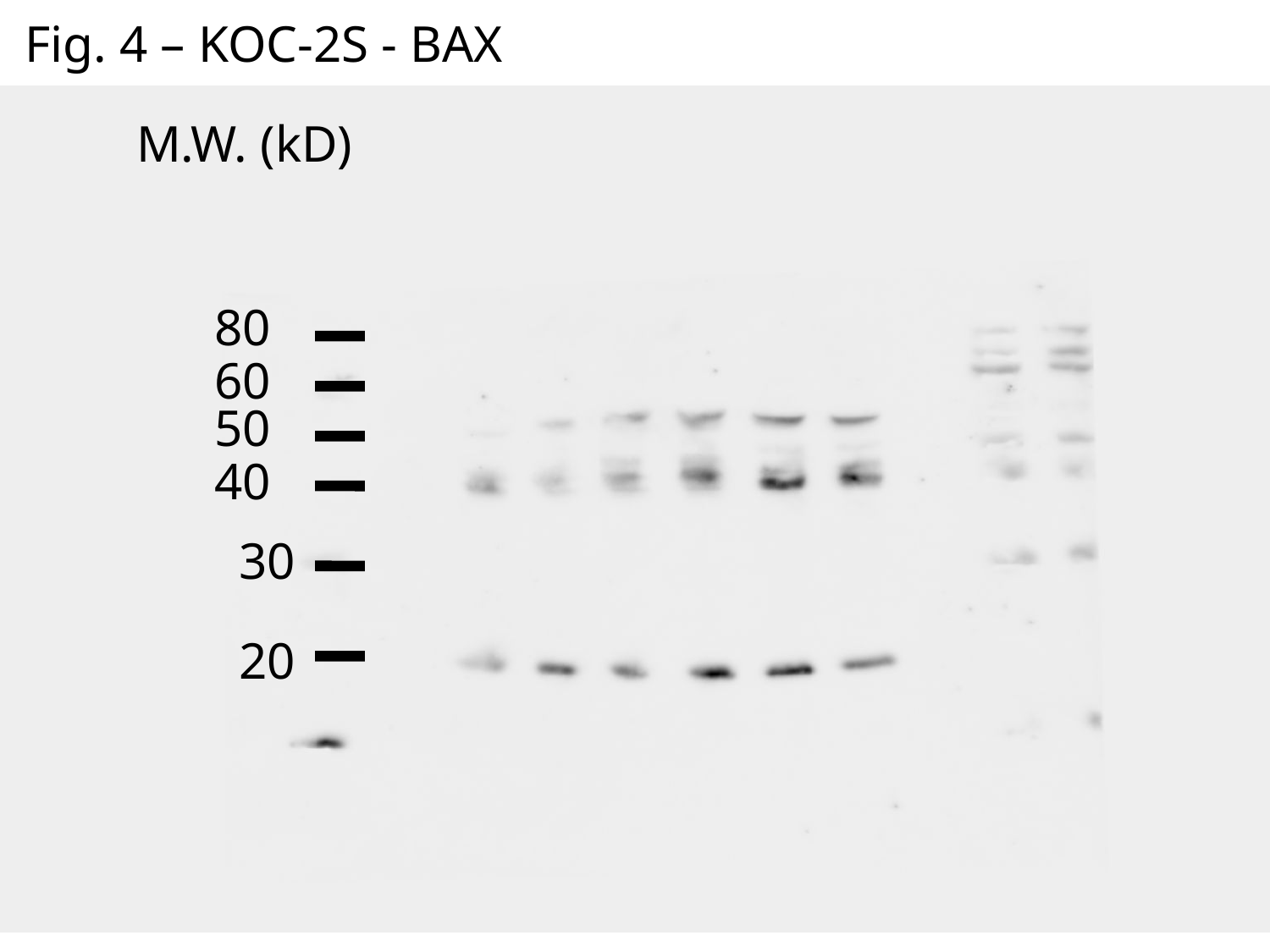

Fig. 4 – KOC-2S - BAX
M.W. (kD)
80
60
50
40
30
20

## Slide 5
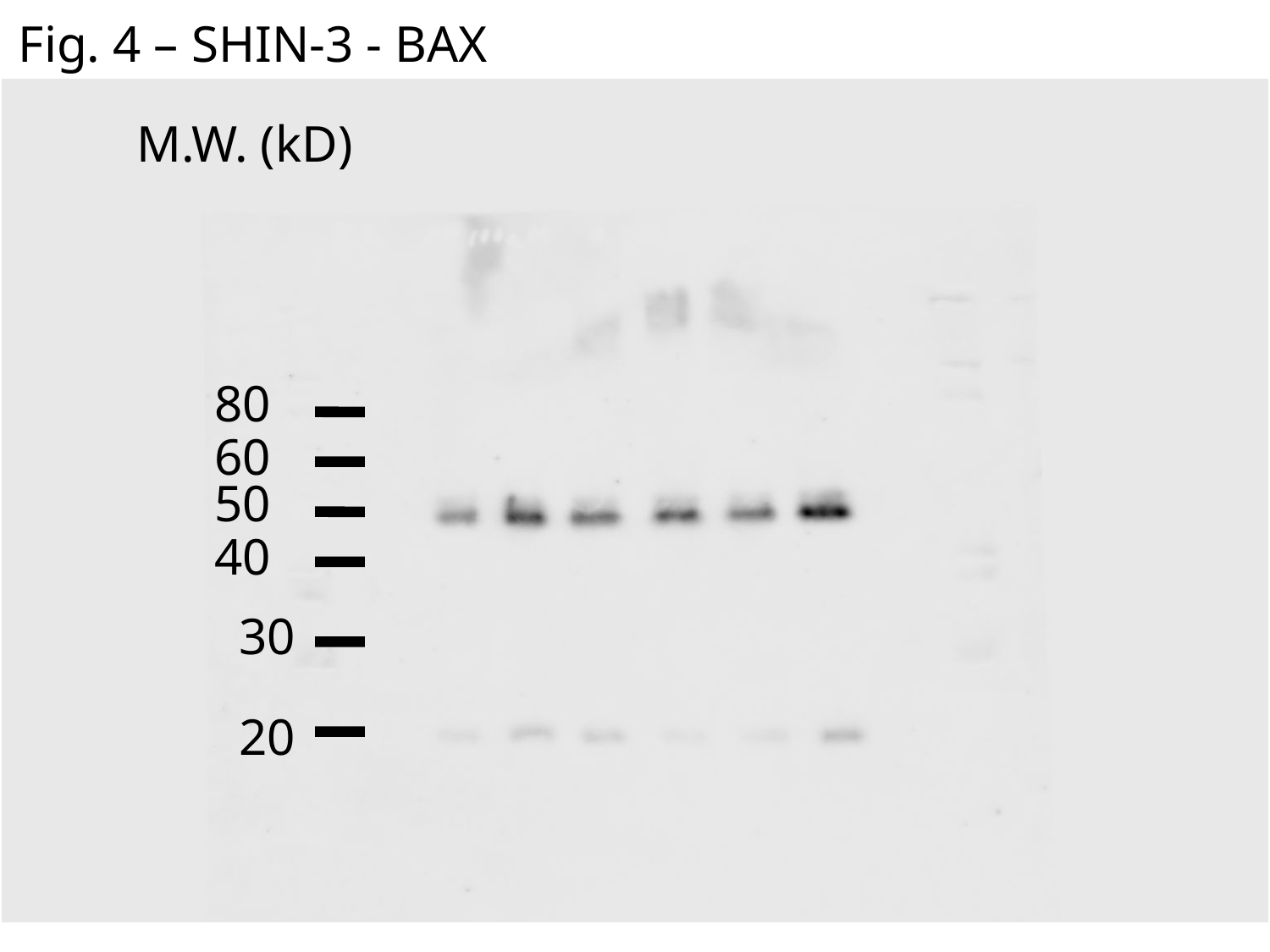

Fig. 4 – SHIN-3 - BAX
M.W. (kD)
80
60
50
40
30
20
